# Dynamic order Markov model for categorical sequence clustering

**DOI:** 10.1186/s40537-021-00547-2

**Published:** 2021-12-07

**Authors:** Rongbo Chen, Haojun Sun, Lifei Chen, Jianfei Zhang, Shengrui Wang

**Affiliations:** 1 Department of Computer Science, University of Sherbrooke, Sherbrooke, Canada; 2 Department of Computer Science, Shantou University, Shantou, China; 3 Department of Computer Science, Fujian Normal University, Fuzhou, China

**Keywords:** Sparse pattern, Pattern detection, Dynamic order Markov model, Categorical sequence clustering

## Abstract

Markov models are extensively used for categorical sequence clustering and classification due to their inherent ability to capture complex chronological dependencies hidden in sequential data. Existing Markov models are based on an implicit assumption that the probability of the next state depends on the preceding context/pattern which is consist of consecutive states. This restriction hampers the models since some patterns, disrupted by noise, may be not frequent enough in a consecutive form, but frequent in a sparse form, which can not make use of the information hidden in the sequential data. A sparse pattern corresponds to a pattern in which one or some of the state(s) between the first and last one in the pattern is/are replaced by wildcard(s) that can be matched by a subset of values in the state set. In this paper, we propose a new model that generalizes the conventional Markov approach making it capable of dealing with the sparse pattern and handling the length of the sparse patterns adaptively, i.e. allowing variable length pattern with variable wildcards. The model, named Dynamic order Markov model (DOMM), allows deriving a new similarity measure between a sequence and a set of sequences/cluster. DOMM builds a sparse pattern from sub-frequent patterns that contain significant statistical information veiled by the noise. To implement DOMM, we propose a sparse pattern detector (SPD) based on the probability suffix tree (PST) capable of discovering both sparse and consecutive patterns, and then we develop a divisive clustering algorithm, named DMSC, for Dynamic order Markov model for categorical sequence clustering. Experimental results on real-world datasets demonstrate the promising performance of the proposed model.

## Introduction

Categorical sequence data have grown enormously in commercial and scientific studies over the past decades. Categorical sequences, such as the biological sequences, web-clicking or behavioral sequences [1–6], are consist of a finite set of categorical values with chronological (or spatial) dependencies that are characteristic of each one of them. For instance, protein sequence is characterized by the 20 possible amino acids with their spatial orders. Discovering and understanding the sequential dependencies hidden in a set of sequences are very important tasks for characterizing sequence families and still pose significant challenges to the field of data mining, in particular, sequence clustering and classification analysis.

Categorical sequence clustering aims at partitioning a set of categorical sequences into homogeneous groups by maximizing their intra-cluster similarity and minimizing their inter-cluster similarity, and has been extensively studied in pattern recognition, web mining, and bioinformatics [7–11]. Many applications have been put into practice by academics and practitioners, such as inferring protein subfamilies [12], predicting biological sequence function [13], discovering behavior patterns from web user profiles [14], and detecting fraud patterns in credit card behaviors [15]. Moreover, sequence clustering helps provide critical insights into the mountains of sequential data without requiring prior knowledge.

The quality of clustering results depends on the similarity measure used to evaluate how well a sequence matches with another sequence or fits a set of sequences/model. Consequently, the biggest challenge in categorical sequence clustering is to define an effective similarity measure between a set of sequences. The existing similarity measures can be classified into two categories: alignment-based and alignment-free methods. The alignment-based methods focus on finding an optimal match by identifying regions shared by sequences, such as the local alignment [16] and global alignment [17], which are often associated with high time complexity or impaired by the presence of statistically significant patterns^1^ in reverse orders. The alignment-free methods, such as the frequent patterns and Markov model based methods [18–20], aim to capture the underlying patterns of the sequences that can be used to represent each sequence (by vectorization) or build a statistic model for clustering analysis. They are in general much more efficient and effective compared to the alignment-based methods for clustering analysis.

**Fig. 1:**
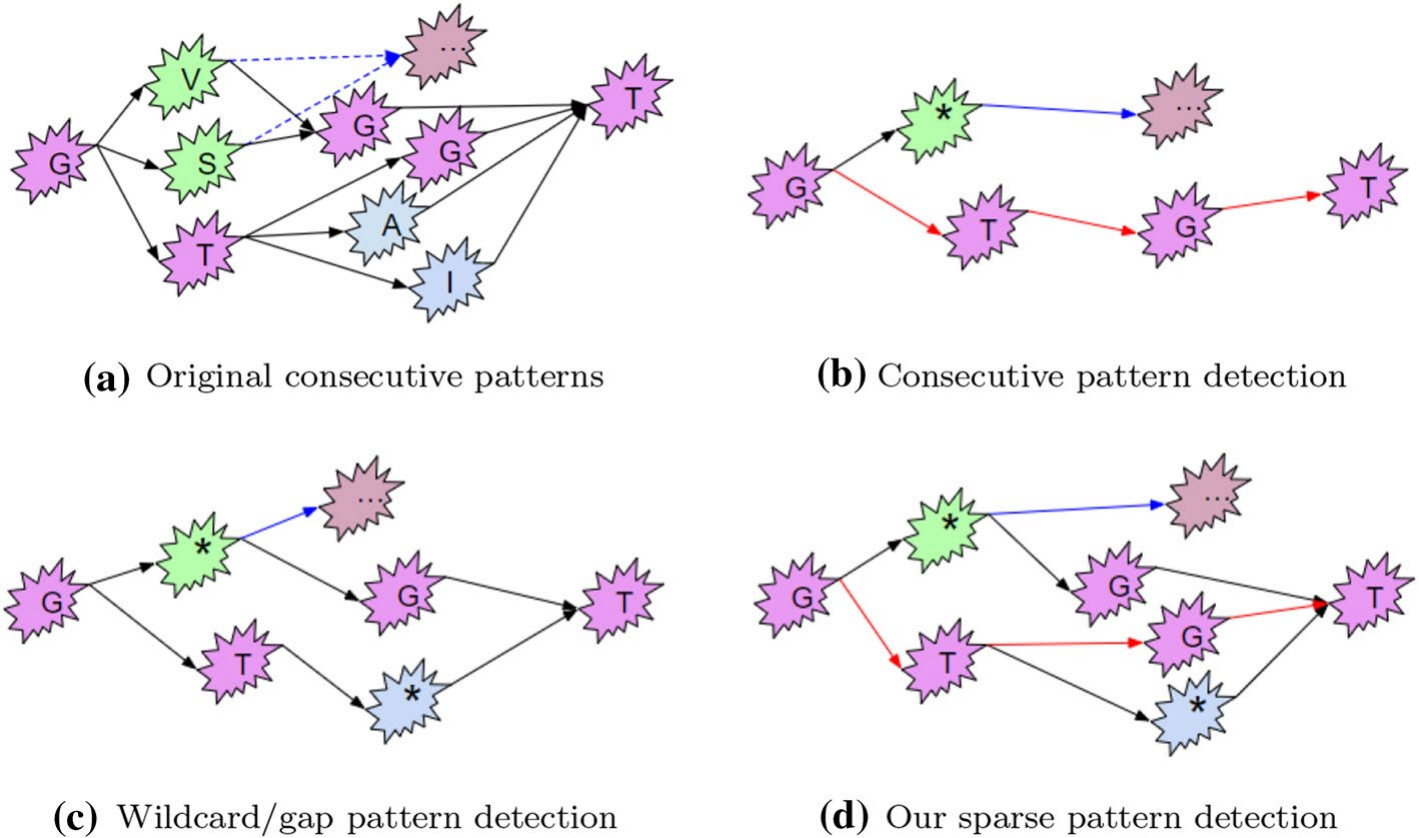
Selected patterns detected from the 3$\beta$-HSD protein family are used here to illustrate the concept of ***sparse pattern***. **a** Shows some of the patterns *GTGT*(25), *GSGT*(16), *GVGT*(18), *GTIT*(17), *GTAT*(15) with occurrence in bracket, which represents that original dataset; **b** Shows patterns detected by conventional consecutive detection method, and only one ***consecutive pattern*** *GTGT*, while the other patterns are filtered by a predefined support threshold $\tau =20$. **c** Shows the patterns detected by the wildcard/gap detection method, yielding only sparse pattern $G*GT$ and $GT*T$ detected since the can not find consecutive patterns. **d** Presents the patterns detected by our approach (sparse pattern detection) with the same threshold. Compared with **b**, **c**, our approach not only detects the sparse patterns $G*GT$ and $GT*T$, but also retains the consecutive pattern *GTGT* as shown in (**d**)

In this paper, we are interested in the methods based on Markov techniques since they have shown to be highly effective in encoding chronological dependencies in sequential data [19, 21, 22]. Generally, such methods proceed in defining a probability framework for capturing the statistically significant patterns hidden in the sequences for sequence representation, while assuming that the probability of next categorical value depends on the proceeding context/pattern^2^. For simplifying computation, the existing methods impose a constraint on each pattern assuming that it is consecutive. This constraint has an effect of preventing exploration of many sub-patterns that are not statistically significant enough in their consecutive form, but could become statistically significant if some *noisy* or *non-frequent values* in the patterns are masked. For instance, the pattern *GTIT* and *GTAT* shown in Fig. 1a are sub-frequent individually, but can become frequent in a sparse form of $GT*T$^3^ in Fig. 1d. More formally, a ***sparse pattern***, such as $G*GT$ and $GT*T$, is a pattern in which one or more of the value/s between the first and last value in the pattern are replaced by wildcard/s that can be matched by a subset of categorical values in the categorical value set. Figure 1d shows that one consecutive pattern *GTGT* and two sparse pattern $G*GT$ and $GT*T$ are discovered in a protein dataset. In this dataset, $G*GT$ is frequent while both patterns *GVGT* and *GSGT* are sub-frequent because of the noisy values *V* and *S*, which is often the case in categorical sequences being imbued with significant quantities of noise and will lead to information loss during the clustering analysis.

A major drawback of conventional Markov model based methods is that they take advantage of only a portion of statistical significant contexts as consecutive pattern detected according to a predefined threshold. All the patterns in Fig. 1a would be frequent while the threshold was set as $\tau =15$, and if we keep all the patterns to make maximal use of information for model learning, which can make use of information underlying in the sequences on the cost of the exponential increase of parameter learning, but also will suffer from the overfitting problem. However, if the threshold were set such as $\tau =20$, only one consecutive pattern would be detected as shown in Fig. 1b, which will cause underfitting problem. ***Overfitting*** or ***Underfitting*** describes sufficiency or insufficiency in the number and content of the patterns detected for the knowledge representation in the discovery process, which may lead to information redundancy or information loss. Although, the wildcard constrained approaches, a well-studied topic, can retain information in the sub-frequent pattern by containing wildcards [23–26], the issue of these methods is that the frequent consecutive patterns will be masked by the sparse patterns. For example, compared with the Fig. 1c, d, the frequent consecutive patterns (GTGT) is covered by the sparse pattern $GT*T$ and $G*GT$, which can not make use of the information in frequent consecutive patterns (GTGT) and may result in underfitting problem. In summary, the existing models likely yield underfitting or overfitting from the model perspective, which will inevitably lead to information loss and has impacts on the performance of clustering analysis, a common limitation of categorical sequence mining.

To best of our knowledge, the existing sequence clustering methods are either focus on detecting the frequent consecutive patterns or sparse patterns for categorical sequence analysis, which results in overfitting or underfitting problem in terms of knowledge discovery and representation. However, no literature reports mining both of them simultaneously for similarity measure for categorical sequence analysis, which promotes us to propose the sparse pattern detector (SPD) to detect both sparse and consecutive pattern for sequence clustering that is a trade-off between underfitting and overfitting for model learning. The basic idea of proposing SPD is to relax the constraint of variable order Markov model on patterns being comprised of consecutive patterns, and this relaxation model is capable of exploiting both sparse and consecutive patterns with variable length, named Dynamic order Markov model (DOMM). In fact, DOMM is a generalization of the Markov approach that can make use of both frequent consecutive pattern and sparse patterns detected from the sub-frequent patterns as shown in Fig. 1d. The major contributions of this work can be summarized as follows:

- We propose a sparse pattern detector (SPD) to discover patterns by using probability suffix tree (PST) with wildcards. SPD not only inherits the capabilities of PST to discover consecutive patterns, but also is capable of discovering sparse patterns from sub-frequent patterns with wildcard, which is completely different from conventional pattern detection methods since it retains the consecutive patterns, and also can detects the sparse patterns from sub-frequent patterns simultaneously.
- We propose a new Dynamic order Markov model that extends conventional Markov model to account for sparse patterns in addition to consecutive patterns, which can take advantage of the statistical information in sub-frequent patterns. Among others, it allows the design of a new similarity measure between a sequence and a cluster.
- We design a cascade algorithm for categorical sequence clustering based on the proposed DOMM, named Dynamic order Markov model for categorical sequence clustering (DMSC). Comprehensive experimental results on real-world datasets demonstrate the promising performance of our algorithm.

The remainder of this paper is organized as follows: we present the related work and preliminaries on Markov model based similarity measures. This is followed by a detailed description of the proposed model, including Dynamic Order Markov Model (DOMM), sparse pattern detector (SPD), the clustering optimizer based on DOMM and the divisive algorithm DMSC; and then we describe the experimental results and analysis on the performance of DMSC. Finally, we present a conclusion of our work and some potential works in the near future.

## Related work and preliminaries

In this section, we will introduce the related work on categorical sequence clustering and the premises of typical Markov chain based models on categorical sequence analysis.

### Categorical sequence clustering

Recent literature reports a number of pattern-based categorical sequence clustering approaches [27–34]. SigClust, proposed by [20], is a signature based clustering method by an n-gram model, which makes use of frequent grams as signature for sequence representation. Ref. [28] proposed an n-gram based method (SCS) that allows mismatches within fixed length frequent consecutive grams to detect shorter grams with variable length for sequence representation. Those approaches suffer, however, from the gram length problem since a fixed length is the cause of either overfitting or pattern underfitting and the optimal length is difficult to obtained. This leads to the development of variable length models [19] and [27] who, in a perspective of pattern exploration, employed the PST to search for frequent and consecutive subsequences (patterns) with maximum-length from a set of sequences and use them as patterns for their Markov chain models. On the other hand, several gap/wildcard constrained methods have been proposed for extracting contexts/patterns for sequential data mining [26, 35–37]. For instance, [36] proposed a sequential pattern mining method using wildcard constraints that are specified by user with flexibility, which needs extra prior insights into the dataset. The model proposed by [37], named SMCD, groups fixed preceding patterns with identical conditional distributions into invariance classes and represents each class by a sparse pattern, which is used as the fixed order of a Markov chain model. Existing gap/wildcard constrained methods address overfitting problem by compressing multiple patterns into a sparse pattern containing wildcards [36], but result in underfitting problem. For instance, in [37], the frequent and consecutive pattern is also included by a sparse pattern ($GTGT \in G*GT$), which is a typical example of underfitting that results in information loss, a common issue for sequential data mining.

### Generative model and similarity measure

The generative Model, widely used to model categorical sequences for clustering and classification, is based on the assumption that the conditional probability distribution of the next categorical value depends on the immediate preceding pattern [14, 21, 27, 37, 38]. For instance, given a sequence $S = s_{1} s_{2}\ldots s_{n}$ of length *n* composed of categorical values from the set $\Sigma$ and a set of sequences denoted by a Markov chain model ${\mathcal {M}}$, the generative probability of the sequence *S* over the model ${\mathcal {M}}$ is defined as follows:

1 $$\begin{aligned} \begin{aligned} P_{{\mathcal {M}}}(S)&= P_{{\mathcal {M}}}(s_{1}) P_{{\mathcal {M}}}(s_{2}|s_{1}) \ldots P_{{\mathcal {M}}}(s_{n}|s_{1}\ldots s_{n-1}) \\&=\prod _{i=1}^{n} P_{{\mathcal {M}}}(s_{i}|s_{1}\ldots s_{i-1}) \end{aligned} \end{aligned}$$

where $s_{0}=\emptyset$ and $P_{{\mathcal {M}}}(s_{1}|s_{0})= P_{{\mathcal {M}}}(s_{1})$ is the frequency of symbol $s_{1}$ over the model ${\mathcal {M}}$, and $P_{{\mathcal {M}}}(s_{i}|s_{1} \ldots s_{i-1})$ is the conditional probability of the next symbol $s_{i}$ right after the preceding pattern $s_{1} \ldots s_{i-1}$ over model ${\mathcal {M}}$. Thereby, $P_{{\mathcal {M}}}(S)$ should be relatively higher, if sequence *S* is subsumed by a more similar model ${\mathcal {M}}$ compared with the other models, which means that $P_{{\mathcal {M}}}(S)$ can be used as the similarity measure between sequence *S* and model ${\mathcal {M}}$ (derived by a set of sequences ${\mathcal {M}}$); namely, the higher the value of $P_{{\mathcal {M}}}(S)$, the more likely that *S* comes from that group of sequences. However, such model requires that all preceding patterns are statistically significant which provides enough statistical information from the model perspective, but it is unrealistic in practice. Thus, the high or variable order Markov model are proposed to provide a better estimation by using constrains on the preceding patterns, yielding the Markov model based similarity measures which are widely used in sequence clustering and classification.

### Markov model

Many Markov model based methods have been proposed for modeling the chronological dependencies of sequential data, which exhibit many interesting characteristics for sequence analysis, such as speech and pattern recognition [39] and sequence clustering [19]. Ref. [21] proposed a higher order Markov chain model for categorical data sequences, which has the potential to capture the chronological dependencies or patterns in sequential data where the dependencies are in a fixed length whose value is set to *L* for simplicity. From the Markov chain model perspective, the high order Markov model can be viewed as an generalization of the Markov chain model, since the pattern $s_{1} \ldots s_{i-1}$ is approximated by the sub-pattern $s_{i-L+1} \ldots s_{i-1}$ with length *L*. Consequently, the *L* order Markov model is defined as follows:

2 $$\begin{aligned} \begin{aligned} P_{{\mathcal {M}}}(s_{i}|s_{1} \ldots s_{i-1}) \approx P_{{\mathcal {M}}}(s_{i}|s_{i-L+1} \ldots s_{i-1}) \end{aligned} \end{aligned}$$

The issue associated with the high order Markov model is that the patterns $s_{i-L+1} \ldots s_{i-1}$ in $s_{i}$ ($1 \le i\le n$) can not all be statistically significant over model ${\mathcal {M}}$ in practice due to the parameter of order *L*. Consequently, they can not provide reliable statistics for for estimating the conditional probability of $s_{i}$, which in turn affects the estimated probability $P_{{\mathcal {M}}}(S)$ from the model perspective. Moreover, the number of model parameters grows exponentially with the orders ($|\Sigma |^{L}(|\Sigma |-1)$ for *L* order). Thus, this model based methods may suffer from overfitting problem. Ref. [40] proposed a SMCD model based on the High order Markov model, which classifies all patterns with length L into several groups shared the same transition probability, such classification can reduce dimension of the parameter space effectively. For instance, there are five pattern *GTGT*, *GTIT*, *GTAT*, *GSGT* and *GVGT* in the Fig. 1a, which can be viewed as a fourth-order Markov chain. The five patterns can be classified into two groups of patterns: {*GSGT*, *GVGT*}, {*GTGT*, *GTIT*, *GTAT*}, which are represented by two sparse patterns $G*GT$ and $GT*T$ respectively as shown in Fig. 1c. However, it is not fair enough for the probability calculation of pattern *GTGT* since it is frequent inherently as shown in Fig. 1d, which will obviously result in underfitting by such representation as aforementioned. Ref. [22] proposed a sequence mining method to represent the higher-order dependencies in networks. Those high order Markov model based methods only can tackle the pattern with fixed length, which may also break chronological dependencies or structurally relevant information that vary in length among sequences.

As aforementioned, the fixed length pattern, to some extent, still can not satisfy the statistical needs of Markov model since any fixed length value may break the chronological pattern dependencies of sequential data. To deal with the pattern with variable length, [41] proposed the variable order Markov model for data compression, which is viewed as a further generalization of Markov chain model. The pattern $s_{1} \ldots s_{i-1}$ is generalized to learn from the given previous pattern that could be varying in length of patterns based on the data being modeled. Ref. [19] proposed a variable order Markov model for categorical sequence clustering by detect only the frequent consecutive pattern by PST for the model representation. Thus, the Eq. (1) is extended to dealing with pattern in variable length as follows:

3 $$\begin{aligned} \begin{aligned} P_{{\mathcal {M}}}(s_{i}|s_{1} \ldots s_{i-1}) \approx P_{{\mathcal {M}}}(s_{i}|s_{j} \ldots s_{i-1}) \end{aligned} \end{aligned}$$

In fact, the pattern $s_{1} \ldots s_{i-1}$ is approximated by the latest frequent sub-pattern $s_{j} \ldots s_{i-1}$ whose length can be larger or smaller than *L* which is capable of capturing the variable chronological dependencies in flexibility. Thus, such approximation can guarantee a reliable statistic for conditional probability estimation accordingly, since if $s_{i-L+1} \ldots s_{i-1}$ is not frequent, $s_{1} \ldots s_{i-1}$ will never be frequent (apriori algorithm), but there exists a sub-pattern $s_{j} \ldots s_{i-1} (j>i-L+1)$ being frequent that avoids making probability approximation meaningless. However, such models suffer from overfitting since they can not deal with the sparse patterns detected from sub-frequent patterns.

## The proposed model

In this section, we will first propose the derivation of Dynamic order Markov model (DOMM) that serves as similarity measure of the sequence clustering, and then describe the sparse pattern detector (SPD), which discovers the sparse and consecutive patterns of DOMM from the categorical sequences. Finally, we will present the divisive algorithm (DMSC) for categorical sequence clustering based on DOMM and SPD.

### Dynamic order Markov model

In light of the above Markov models, we propose a new Dynamic order Markov model (DOMM) that is capable of modeling the sparse and consecutive patterns of variable length, including consecutive pattern (*GTGT*) and sparse pattern ($GT*T$, $G*GT$) shown in Fig. 1d, in order to solve the challenges of the conventional Markov models. According to the Markov chain model, we therefore define the new DOMM in a general form as follows:

4 $$\begin{aligned} \begin{aligned} P_{{\mathcal {M}}}(s_{i}|s_{1} \ldots s_{i-1}) \approx P_{{\mathcal {M}}}(s_{i}|s_{\psi _{m}}w^{\phi _{m}} \ldots s_{\psi _{2}}w^{\phi _{2}} s_{\psi _{1}}w^{\phi _{1}}) \end{aligned} \end{aligned}$$

where $\psi _{m}=i-m-\sum _{j=0}^{m}\phi _{j}$, $s_{\psi }$ denotes the $\psi{\text{th}}$ categorical value of the pattern $s_{1} \ldots s_{i-1}$, while $w^{\phi }$ denotes $\phi$ consecutive wildcards ‘*’. Note that the model in Eq. (4) will decay to the variable order Markov model in Eq. (3) if $\phi _{j}=0$ for all *j*. $P_{{\mathcal {M}}}(s_{i}|s_{\psi _{m}}w^{\phi _{m}} \ldots s_{\psi _{2}}w^{\phi _{2}} s_{\psi _{1}}w^{\phi _{1}})$ is the conditional probability of symbol $s_{i} (s_{i} \in \Sigma )$ occurring right after the preceding pattern (consecutive or sparse pattern) $s_{\psi _{m}}w^{\phi _{m}} \ldots s_{\psi _{2}}w^{\phi _{2}} s_{\psi _{1}}w^{\phi _{1}}$ denoted by $\theta _{i}$. Thereby, $P_{{\mathcal {M}}}(s_{i}|s_{\psi _{m}}w^{\phi _{m}} \ldots s_{\psi _{2}}w^{\phi _{2}} s_{\psi _{1}}w^{\phi _{1}})$ in Eq. (4) is simplified to $P_{{\mathcal {M}}}(s_{i}|\theta _{i})$. Statistical information in this paper denotes the number of sequences in corresponding group or cluster, the length of each sequence and the pattern occurrence in each sequence. In order to make use of these statistical information in sequence *S* and model ${\mathcal {M}}$, the probability $P_{{\mathcal {M}}}(s_{i}|\theta _{i})$ in Eq. (4) is divided into two part: one part is from sequence S: ${\hat{P}}_{S}(s_{i}|\theta _{i})$, the other is from model ${\mathcal {M}}$: ${\hat{P}}_{{\mathcal {M}}}(s_{i}|\theta _{i})$. We therefore redefined model probability $P_{{\mathcal {M}}}(s_{i}|\theta _{i})$ in Eq. (4) as follows:

5 $$\begin{aligned} P_{{\mathcal {M}}}(s_{i}|\theta _{i}) = \exp \left( -\frac{\left( {\hat{P}}_{S}(s_{i}|\theta _{i})-{\hat{P}}_{{\mathcal {M}}}(s_{i}|\theta _{i})\right) ^2}{{\hat{P}}_{{\mathcal {M}}}(s_{i}|\theta _{i})}\right) \end{aligned}$$

where ${\hat{P}}_{S}(s_{i}|\theta _{i})$ is the conditional probability of $s_{i}$ given the preceding pattern $\theta _{i}$ in sequence *S*, and ${\hat{P}}_{{\mathcal {M}}}(s_{i}| \theta _{i})$ is referred to as the model parameter derived from cluster *C*, which will be inferred later. If sequence *S* coming from model *M*, the conditional probability distribution of $s_{i}$ right after pattern $\theta _{i}$ in sequence *S* will be the same as model ${\mathcal {M}}$ and the $\exp (\cdot )$ is equal to 0, thus, $P_{{\mathcal {M}}}(s_{i}|\theta _{i})$ would be equal to 1; otherwise, the $\exp (\cdot )$ is equal to $-\infty$, and $P_{{\mathcal {M}}}(s_{i}|\theta _{i})$ would be approaching but not equal to 0. Thus, we guaranteed that the probability $P_{{\mathcal {M}}}(s_{i}|\theta _{i})$ is in the range 0 to 1.0. To take the sequence length and pattern occurrence into account, we estimate ${\hat{P}}_{S}(s_{i}|\theta _{i})$ as follows:

6 $$\begin{aligned} \begin{aligned} {\hat{P}}_{S}(s_{i}|\theta _{i})&= P_{S}(s_{i}|\theta _{i})\times \omega _{S}^{\theta _{i}} \end{aligned} \end{aligned}$$

where $P_{S}(s_{i}|\theta _{i})=\frac{n(\theta _{i}s_{i},S)}{n(\theta _{i},S)}$ is the experience probability of $s_{i}$ following the pattern $\theta _{i}$ in sequence *S*, while $\omega _{S}^{\theta _{i}} = \frac{n(\theta _{i},S)}{|S|}$ is the frequency of $\theta _{i}$ occurs in *S*; $n(\theta _{i}s_{i},S)$, $n(\theta _{i},S)$ denote the number of occurrence of pattern $\theta _{i}s_{i}$ and $\theta _{i}$, respectively, in sequence *S*.

For the occurrence calculation as $n(\theta _{i}s_{i},S)$ in Eq. (6), if the pattern $\theta _{i}$ is consecutive, it is easy to calculate ${\hat{P}}_{S}(s_{i}|\theta _{i})$ by the occurrence of $\theta _{i}s_{i}$ and sequence length |*S*|. However, if $\theta _{i}$ is a sparse pattern with wildcard/s whose number and position varies among different pattern. We assume that there is one wildcard (denoted by *) in pattern $\theta _{i}$ for simplicity, so that the pattern $\theta _{i}$ can be denoted as $\theta _{1}*\theta _{2}$ ($\theta _{1}$ and $\theta _{2}$ being the consecutive parts of sparse pattern $\theta _{i}$). Thereby, $n(\theta _{i}s_{i},S)$ and $n(\theta _{i},S)$ in Eq. (6) can be calculated as follows:

7 $$\begin{aligned} \begin{aligned} n(\theta _{i}s_{i},S) = \sum _{q \in V} n(\theta _{1}q\theta _{2} s_{i},S) \\ n(\theta _{i},S) = \sum _{q\in V} n(\theta _{1}q\theta _{2} ,S) \end{aligned} \end{aligned}$$

Thus, according to the Markov chain model in Eq. (1), and the DOMM in Eq. (5), the probability of sequence *S* coming from model ${\mathcal {M}}$ can be redefined as follows:

8 $$\begin{aligned} \begin{aligned} P_{{\mathcal {M}}}(S)&=\prod _{i=1}^{|S|} P_{{\mathcal {M}}}(s_{i}|\theta _{i})\\&=\prod _{i=1}^{|S|} \exp \left( -\frac{\left( {\hat{P}}_{S}(s_{i}|\theta _{i})-{\hat{P}}_{{\mathcal {M}}}(s_{i}|\theta _{i})\right) ^2}{{\hat{P}}_{{\mathcal {M}}}(s_{i}|\theta _{i})}\right) \end{aligned} \end{aligned}$$

where ${\mathcal {M}}$ is derived from a set of sequences (refered to as cluster *C*), the larger $P_{{\mathcal {M}}}(S)$, the more likelihood of sequence *S* coming from cluster *C*. Thus, this generative probability can be similarity measurement between a sequence and a cluster. According to the principle of relativity for similarity measures, we omitted exponential function in Eq. (8) to simplify calculation, and defined a new DOMM based similarity measurement as follows:

9 $$\begin{aligned} sim(S,C) = -\sum _{i=1}^{|S|} \frac{\left( {\hat{P}}_{S}(s_{i}|\theta _{i})-{\hat{P}}_{{\mathcal {M}}}(s_{i}|\theta _{i})\right) ^2}{{\hat{P}}_{{\mathcal {M}}}(s_{i}|\theta _{i})} \end{aligned}$$

To be consistent with the conventional similarity measures, we turn the similarity measure in Eq. (9), into a dissimilarity measure (dsim) as follows:

10 $$\begin{aligned} dsim(S,C) = \sum _{i=1}^{|S|} \frac{\left( {\hat{P}}_{S}(s_{i}|\theta _{i})-{\hat{P}}_{{\mathcal {M}}}(s_{i}|\theta _{i})\right) ^2}{{\hat{P}}_{{\mathcal {M}}}(s_{i}|\theta _{i})} \end{aligned}$$

As is clear from Eq. (10), the dissimilarity measure *dsim*(*S*, *C*) is meaningless when *i* is equal to 1 as $s_{1}$ has no preceding pattern. However, there are two issue during the Eq. (10) calculation: one is that $s_{i}$ will be repeated multiple times in *S* and map to the same pattern, another is that there are many short patterns detected by SPD. Thus, we ignore the short patterns and use all the longest frequent patterns to represent the sequences as for the dissimilarity approximation. Therefore, the dissimilarity approximation in Eq. (10) can be rewritten as follows:

11 $$\begin{aligned} \begin{aligned} d(S,C)&= \sum _{s \in \Sigma } \sum _{\theta \in \Theta } \frac{\left( {\hat{P}}_{S}(s|\theta )-{\hat{P}}_{{\mathcal {M}}}(s|\theta )\right) ^2}{{\hat{P}}_{{\mathcal {M}}}(s|\theta )}\\ \end{aligned} \end{aligned}$$

where $\Theta$ ($\theta \in \Theta$), $\Sigma$ ($s \in \Sigma$) denotes all the longest frequent pattern detected by SPD in cluster *C* and the distinct categorical values that consist of the whole sequence in cluster *C*. We thus can use the dissimilarity measure in Eq. (11) to refine clusters by relocating sequence and estimate the compactness of a cluster. Based on Eq. (10), we can therefore define the clustering objective function of a categorical sequence dataset with *K* clusters. The objective function for the Sum of Clustering Error (SCError) is given as follows:

12 $$\begin{aligned} SCError = \sum _{k=1}^{K} \sum _{S \in C_{k}}d(S,C) \end{aligned}$$

By minimizing the objective function Eq. (12) as presented in [42], we can obtain the parameter ${\hat{P}}_{{\mathcal {M}}}(s_{i}|\theta _{i})$ of model ${\mathcal {M}}$ derived from the corresponding cluster as follows:

13 $$\begin{aligned} {\hat{P}}_{{\mathcal {M}}}(s_{i}|\theta _{i}) = \left( \frac{\sum _{S \in C}|S|{\hat{P}}^{2}_{S}(s_{i}|\theta _{i})}{\sum _{S \in C}|S|}\right) ^{\frac{1}{2}} \end{aligned}$$

### Sparse pattern detector

**Fig. 2:**
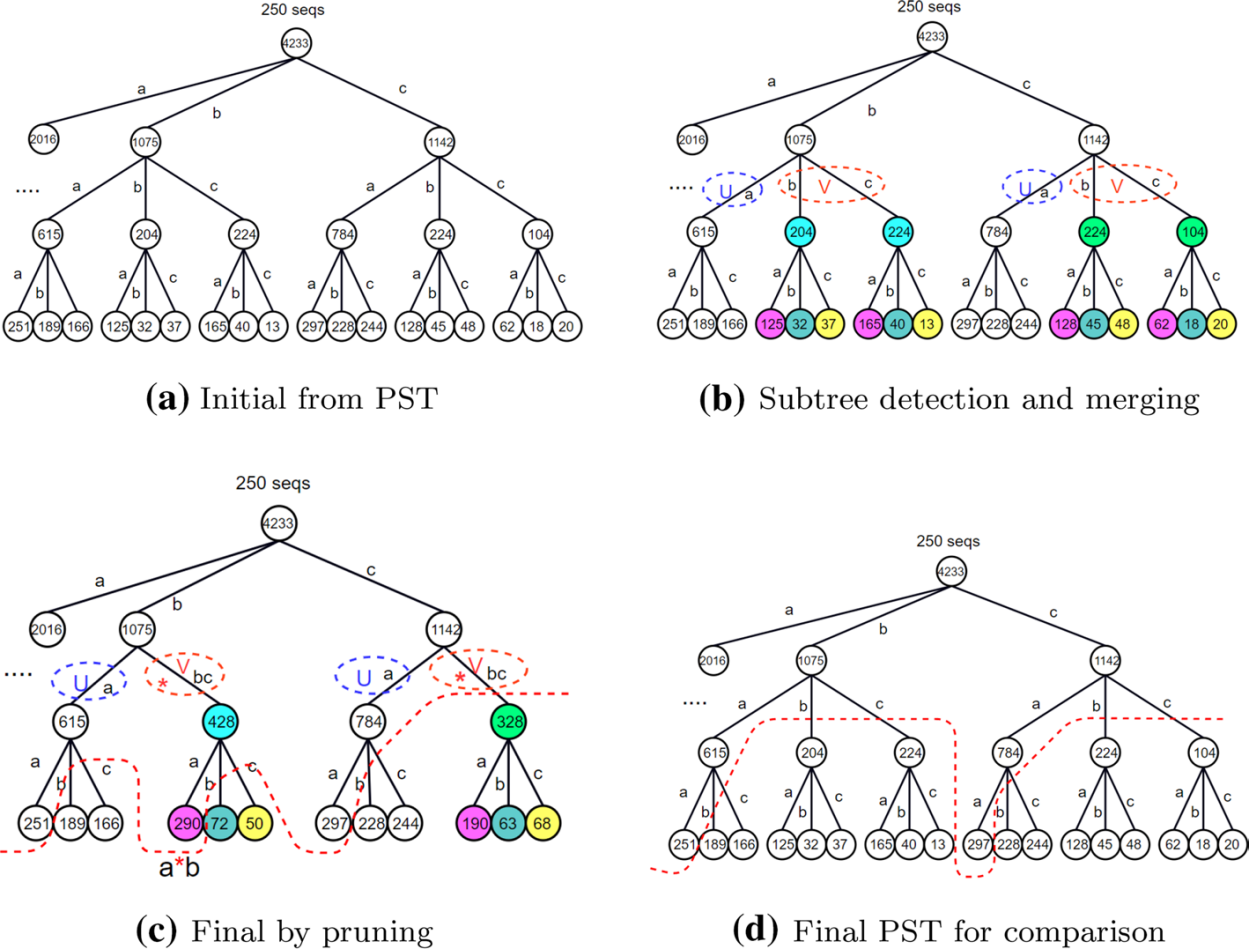
To compare with the PTS above, we use the same dataset (250 sequences over $\Sigma = \lbrace { a, b, c \rbrace }$ L = 3 and $\tau$ = 250) to demonstrate the pattern detection process of SPD: **a** Initial PST constructed from dataset without pruning. **b** Find merge candidates with threshold $\tau$, the subtrees share the same color will be merged. **c** Final SPD, the node below red dotted line will be pruned. **d** Final PST with threshold $\tau =250$ for comparison with SPD. There is an extra sparse pattern $\mathbf {a*b}$ in (**c**) compared with (**d**)

The sparse pattern detector (SPD) is used to detect the sparse and consecutive pattern for DOMM. The intuition behind the SPD is derived from the framework of PST containing wildcards that not only inherits capability of PST detecting the frequent consecutive patterns, but also exploits sparse pattern from the sub-frequent patterns, which can make use of the statistical information in the form of sparse pattern. To provide an illustrative building process of SPD, we decompose the building process into three steps for a better understanding that happens simultaneously in practice. We start by building a PST on a cluster *C* with 250 sequences over $\Sigma = \lbrace {a, b, c\rbrace }$ without pruning as shown in Fig. 2a. For simplicity, we set the maximum depth to *L* = 3 and the threshold to $\tau =250$, while the depth is not limited in actuality, but we will be set to a maximal depth for the sake of space complexity in practice.

Then, we shall select those nodes with occurrence under the support threshold $\tau$ to merge to retain the frequent consecutive patterns, and identify the sparse patterns from sub-frequent consecutive ones. We thus need to scan the number of occurrences at each node and compare it to the threshold $\tau$, starting from the root. If the number of occurrences is smaller than $\tau$, the node will be pruned, since its descendants’ occurrence will never be greater than $\tau$ according to Apriori algorithm; otherwise the children of that node are classified into two groups: *U* (occurrence greater than $\tau$) and *V* (occurrence less than $\tau$). Technically, $U \cup V=\Sigma$, $\lbrace {a\rbrace } \in U$ and $\lbrace {b, c\rbrace } \in V$) as shown in Fig. 2b. Then, we keep the node in *U* and merge the node in *V* if necessary. Actually, the node merging process is subtrees merging, since each node in *V* can be regarded as the root of a subtree. Thus, we merge all the subtrees in *V* with their descendants, and then update the occurrences at each node and label the edge by $*$. For instance, the two subtrees in blue and the two in green in Fig. 2b will be merged to yield the results shown in Fig. 2c.

Finally, we obtain the final SPD by node pruning. We compare the number of occurrences in each children of the merged subtree with the threshold $\tau$ in order to determine whether or not any sparse pattern has been detected after the node merging process. If the number of occurrences at the root of the merged subtree is still below the threshold $\tau$ and no new child with occurrence above $\tau$ has been produced, the merged subtree will be pruned (subtree in green in Fig. 2c; otherwise the merged subtree is kept and the edge is labeled by $*$ (new child, pink node, with occurrence 290 in Fig. 2c, and the final SPD is shown in Fig. 2d. However, the parameter $\tau$ for construction here is simply set to the number of sequences in the corresponding cluster, which is based on the frequent pattern assumption that a frequent pattern should occur at least once in each sequence in the cluster. The thresholds change dynamically according to the number of sequences in each clusters during the iterative process of clustering refinement, which can overcome the hindrance to pattern detection caused by a fixed threshold for all clusters since some patterns may be only frequent in one cluster, but not necessary frequent in another. Moreover, if we use the same fixed threshold for all clusters, most of the patterns may be filtered by the same threshold, especially for the unbalance clusters. The construction process of SPD is given in Algorithm 1: 
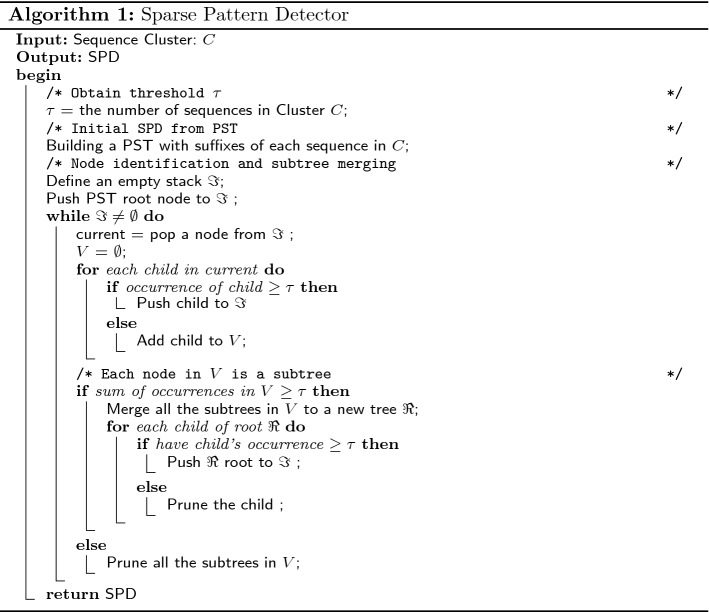


### Clustering optimizer

In this subsection, we design a clustering optimizer to refine the clusters based on the DOMM. The procedure of clustering optimizer can be divided into three steps: firstly, we need to build a SPD on each cluster, which is used to detect the sparse and consecutive pattern for DOMM, and then we update the model parameters of each cluster based on the patterns detected by SPD from each clusters. Finally, we relocate sequences to clusters iteratively based on the dissimilarity measure Eq. (11) until no membership changes. The process of the clustering optimizer are detailed in Algorithm 2. 
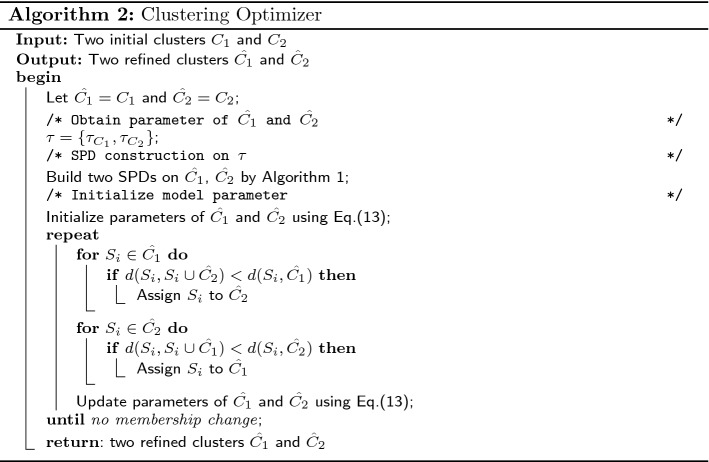


### The DMSC algorithm

**Fig. 3:**
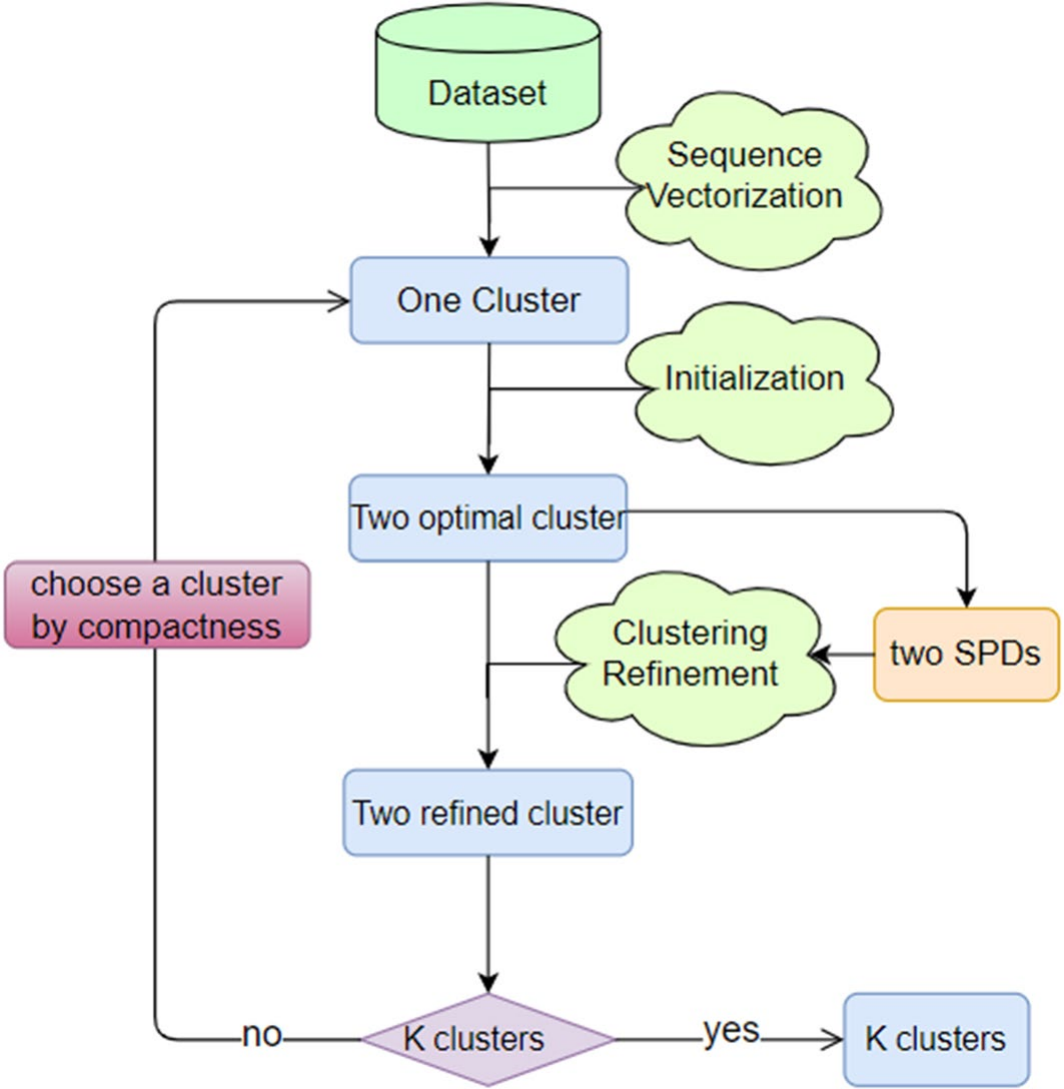
The overview of DMSC consists of the sequence vectorization and an iterative process, while the iterative process includes initialization and clustering refinement

In this subsection, we describe the divisive algorithm of DMSC in details. The process of DMSC involves the sequence vectorization and an iterative phase. The sequence vectorization is to transform each sequence in the dataset into a fixed length vector by the first order Markov chain. The iterative phase includes two steps: initialization and clustering refinement. The initialization includes bisecting one cluster in two initial clusters based on the sequence vector matrix obtained from sequence vectorization phase, and optimizing them according to Chi-square distance. The clustering refinement step refines the two optimized clusters by the clustering optimizer shown in the Algorithm 2 in “Clustering optimizer” subsection, and then choose one cluster for the next iteration until the predefined K clusters achieved. The dissimilarity measure in Eq. (11) can also be used as the estimation of compactness for a cluster, we thus can choose the cluster with maximal compactness for the bisecting process in the initialization phase. The overview of DMSC is shown in Fig. 3.

#### Sequence vectorization

In this phase, we use the first order Markov chain model to transform each categorical sequence into a numerical vector with fixed length ($|\Sigma |^{2}$), which is referred to as Markov Vector of Sequence ($\mathbf {MVS}$). Thus, the sequence set will be converted into a sequence $\mathbf {MVS}$ matrix that will be used for initialization. As example for the transformation process of a sequence to an $\mathbf {MVS}$, we select a sequence $S=ababababbcbabcabacbacbacacaa$ ($\Sigma = \{a, b, c\}$ and $|\Sigma | = 3$) from the previous dataset whose length *n* is 28. The symbol frequency vector $\mathbf {V_{F}}$ and transition occurrence matrix $M_{O}$ are obtained from the frequency of symbol and the state transition by the first order Markov model from sequence *S* respectively, which are shown as follows:

$$\begin{aligned} \mathbf {V_{F}}&= \begin{bmatrix} F_{S_{a}} &{} F_{S_{b}} &{} F_{S_{c}} \\ \end{bmatrix} = \begin{bmatrix} 0.500 &{} 0.286 &{} 0.214 \\ \end{bmatrix}\\ M_{O}&= \begin{bmatrix} O_{S_{aa}} &{} O_{S_{ab}} &{} O_{S_{ac}} \\ O_{S_{ba}} &{} O_{S_{bb}} &{} O_{S_{bc}} \\ O_{S_{ca}} &{} O_{S_{cb}} &{} O_{S_{cc}} \end{bmatrix}= \begin{bmatrix} 3 &{} 5 &{} 5 \\ 7 &{} 0 &{} 1 \\ 3 &{} 3 &{} 0 \\ \end{bmatrix} \end{aligned}$$

where $F_{S_{a}}$ is the frequency of *a* in sequence *S*, and $O_{S_{ij}}$ is the occurrence of state transition from *i* to *j* in sequence *S*. However, the occurrence of observed state transition shown in matrix $M_{O}$ is not fair enough to serve as the representation for a stochastic Markov process of a categorical sequence. We thus introduce the Bayesian approach to model the transition probability among states, other than just use the state transition occurrence dividing by total occurrence, which can guarantee the stochastic process of a Markov model (for more details, please read the reference [43]). According to the Bayesian estimation, the state transition probability thus can be defined as follows:

14 $$\begin{aligned} p_{ij} = \frac{\eta _{ij}}{\eta _{i}} \frac{\eta _{i}}{\eta _{i}+O_{i}} +\frac{O_{ij}}{\eta _{i}+O_{i}} \end{aligned}$$

where $\eta _{ij}$ is a hyper-parameter and $O_{ij}$ is the state transition occurrence of $s_{j}$ given $s_{i}$, $\eta _{i} = \sum _{j} \eta _{ij}$, $f_{i} = \sum _{j} O_{ij}$, $\eta = |\Sigma |$ and $\eta _{ij} = \frac{1}{|\Sigma |}$. Thus, the occurrence transition matrix $M_{O}$ can be transformed into the state transition probability as $M_{P}$ by Bayesian estimation in Eq. (14) as follows:

$$\begin{aligned} M_{P}&= \begin{bmatrix} P_{O_{S_{aa}}} &{} P_{O_{S_{ab}}} &{} P_{O_{S_{ac}}} \\ P_{O_{S_{bb}}} &{} P_{O_{S_{ba}}} &{} P_{O_{S_{bc}}} \\ P_{O_{S_{ca}}} &{} P_{O_{S_{cb}}} &{} P_{O_{S_{cc}}} \end{bmatrix} = \begin{bmatrix} 0.238 &{} 0.381 &{} 0.381 \\ 0.815 &{} 0.037 &{} 0.148 \\ 0.476 &{} 0.476 &{} 0.048 \\ \end{bmatrix} \end{aligned}$$

Using the symbol frequency as weight, the state probability transition can be further estimated as $M_{FP}$ by the state probability transition $M_{P}$ and symbol frequency $\mathbf {V_{F}}$ as follows:

$$\begin{aligned} M_{FP}&=Diag(\mathbf {V_{F}})M_{P}\\&= \begin{bmatrix} F_{S_{a}} &{} &{} \\ &{} F_{S_{b}} &{} \\ &{} &{} F_{S_{c}}\\ \end{bmatrix} \begin{bmatrix} P_{O_{S_{aa}}} &{} P_{O_{S_{ab}}} &{} P_{O_{S_{ac}}} \\ P_{O_{S_{bb}}} &{} P_{O_{S_{ba}}} &{} P_{O_{S_{bc}}} \\ P_{O_{S_{ca}}} &{} P_{O_{S_{cb}}} &{} P_{O_{S_{cc}}} \end{bmatrix}\\&= \begin{bmatrix} 0.500 &{} &{} \\ &{} 0.286 &{} \\ &{} &{} 0.214\\ \end{bmatrix} \begin{bmatrix} 0.238 &{} 0.381 &{} 0.381 \\ 0.815 &{} 0.037 &{} 0.148 \\ 0.476 &{} 0.476 &{} 0.048 \\ \end{bmatrix}\\&= \begin{bmatrix} 0.119 &{}0.1905 &{} 0.1905\\ 0.233 &{}0.011 &{}0.042\\ 0.102&{}0.102&{}0.010 \\ \end{bmatrix} \end{aligned}$$

Finally, sequence *S* is transformed into an $\mathbf {MVS}$ by flattening the state probability transition matrix $M_{FP}$ into one dimension vector as follows:

$$\begin{aligned} \mathbf {MVS}= \begin{bmatrix} 0.119 &{}0.1905 &{} 0.1905&{} 0.233 &{}0.011 &{}0.042&{}0.102&{}0.102&{}0.010 \\ \end{bmatrix} \end{aligned}$$

Therefore, any sequence set can be transformed into a sequence vector matrix composed of the $\mathbf {MVS}$ of each sequence, which can be used for cluster splitting and optimization in the initialization phase.

#### Initialization

In this phase, we need to bisect one cluster (initial dataset denoted as one cluster), and optimize the two clusters according to the Chi-square distance. They will be used as input for further optimization in the phase of clustering refinement. As presented above, each sequence in the cluster has been converted into a sequence $\mathbf {MVS}$. However, such $\mathbf {MVS}$ is highly sparse in dimensionality as 9 dimensions with $\Sigma =3$ and 400 dimension for a protein sequence ($\Sigma =20$). We therefore utilize the principal component analysis (PCA) to reduce the dimensionality on the $\mathbf {MVS}$ matrix of cluster. To obtain two initial robust clusters with maximal distance, we take first component of PCA, explains the largest portion of the variance on the sequence matrix, to bisect the cluster into two initial clusters, namely, sequence with positive value in first component will be assigned to one cluster; otherwise it will be assigned to the other. We then obtain two initial clusters and use the Chi-square distance to optimize the two initial clusters on their $\mathbf {MVS}$ to obtain two robust optimal $C_{1}$ and $C_{2}$ for clustering refinement.

#### Clustering refinement

In this phase, we need to refine the two optimal clusters from the initialization phase. Since we have obtained two optimal clusters $C_{1}$ and $C_{2}$ in initialization phase, and then we can obtain the parameter $\tau$ for SPD construction whose value is equal to the number of sequences in the corresponding cluster as discussed before. Thus, two SPDs will be built on $C_{1}$ and $C_{2}$, which serves as the sparse and consecutive pattern detector shown in Algorithm 1 for the similarity measure in the clustering optimizer in Algorithm 2. The clustering refinement is an iterative process that operates by reassigning sequences to the cluster with minimal dissimilarity measure in Eq. (11). After the clustering refinement, as aforementioned, we need to choose one cluster to repeat for the next iteration until the desired K clusters obtained. The procedure of algorithm DMSC is shown in Algorithm 3.
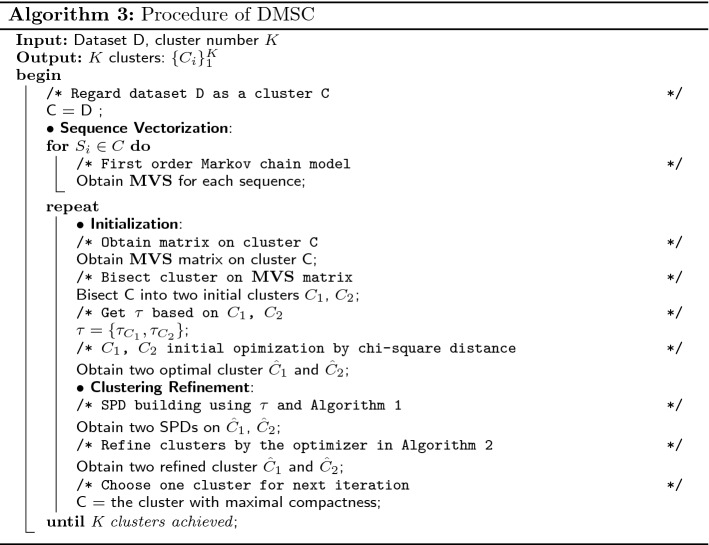


## Experiment

In this section, we will compare the results of our model with the state-of-the-art algorithms on six real-world datasets, and evaluate its performance based on the empirical results.

### Datasets

Clustering biological sequences poses significant challenges for biological sequence analysis in bioinformatics, we therefore choose two biological sequence datasets, one speech sequence dataset and other three datasets to evaluate the performance of our model. The details of the six datasets are shown as follows:

- Protein: This dataset contains 2074 protein sequences from three families, which is available from the SWISS-PROT protein sequence data bank. These sequences are arbitrarily chosen from the globin, immunoglobulin, and 3$\beta$-HSD families, with respective sizes of 1500, 313 and 261. Sequence lengths vary from 10 to 1709, with an average length of 310.
- RNA: This dataset comprises three classes of RNA sequence from RFAM database of non-coding RNA families: transfer messenger RNA (abbreviated tmRNA), signal recognition particle RNA (SRP) and transfer RNA (tRNA). The respective sizes of classes are 390, 254 and 640 sequences. Sequence lengths vary from 54 to 380, and the average length is 201.
- Speech: This dataset includes 500 sequences from five classes (100 sequences for each class). All the sequences were generated from the pronunciation of five isolated French vowels (i.e., ‘a’, ‘e’, ‘i’, ‘o’, ‘u’) and each pronunciation is characterized with a categorical sequence with 20 different categorical values [44]. Sequence lengths vary from 701 to 3753, with an average length being 1898.
- Robot: This dataset consists of two classes: Move-Forward with 2205 samples and SharpRight-Turn with 2097 samples. Each sequence consists of ultrasound readings collected by the sensors of a robot, and is discretised into 95 categorical values, which is used by [45]. Sequence lengths vary from 4 to 100, with an average length being 40.
- Pioneer: this dataset is derived from the pioneer-1 dataset in UCI repository. The time series is manually discretized into three bins (referred to as gripper, move, or turn) based on exploratory data analysis. The numerical time series thus can be categorized by one of three scenarios: gripper, move, or turn [46]. Each sequence is with the same lengths being 24.
- Pattern: this dataset is also comporsed of time interval sequence converted into categorical sequence based on exploratory data analysis. Each time interval in the sequence is characterized by one of five categorical value such as street or meeting [47]. Sequence lengths vary from 22 to 246, with an average length being 88.

The statistics of the datasets are shown in Table 1.

**Table 1:** Dataset description

Dataset	Size	Categorical value	Classes	Length.range	Length.average
Protein	2074	20	3	10–1709	232
RNA	1284	4	3	54–380	201
Speech	500	20	5	179–3753	1101
Robot	4302	95	2	24–24	24
Pioneer	160	178	3	4–100	40
Pattern	240	94	5	22–246	88

### Baselines

To demonstrate the performance of DMSC, we present our results in comparison with following baselines on the six test datasets. The description of the baselines is as follows:

- SigClust [20] is a n-gram based approach, in which each sequence is represented by a binary document signature vector, and uses k-means for clustering on the sequence vector matrix.
- CLUSEQ [27] is a Markov chain model and PST-based method, which uses PST to capture the frequent consecutive patterns as entities of the conditional pattern for the similarity calculation.
- SCS [28] is a variation of n-gram that allows mismatches between patterns within length *L* by detecting the frequent consecutive patterns with variable length for sequence representation.
- WCPD [19] is also a variable order Markov model, which employs the PST to detect consecutive, statistically significant pattern for similarity estimation and model representation.
- SMCD [37] is a fixed Markov chain model for sequence classification, utilizes Delaunay triangulation to cluster the possible preceding pattern in invariance classes with identical conditional distributions, but here we modify it as a sequence clustering method by using the entites of a fixed Markov model to predict the next categorical value for the generative probability of a given sequence.

### Performance metrics

As for the performance metric, besides accuracy, we also employ the F1-measure and Normalized Mutual Information (NMI) to evaluate the quality of the clustering results. They are extensively used metrics for evaluating the performance of clustering analysis [48]. Given two sets of the cluster results ${\mathcal {C}}$ = {$C_{1},\ldots ,C_{K}$} and the ground truth ${\mathcal {G}}$ = {$G_{1},\ldots ,G_{K}$}, the accuracy, F1-measure and NMI are defined respectively as follows:

15 $$\begin{aligned} Accuracy= & {} \frac{\sum _{k=1}^{K} \sum _{S\in C_{k}}1\left\{L_{S}={\mathcal {L}}_{S}\right\}}{N} \end{aligned}$$

16 $$\begin{aligned} F1{\text{-}}measure =\frac{2 \times precision \times recall}{precision + recall} \end{aligned}$$

17 $$\begin{aligned} NMI({\mathcal {C}},{\mathcal {G}})= & {} \frac{2 \times I({\mathcal {C}},{\mathcal {G}})}{H({\mathcal {C}})+H({\mathcal {G}})} \end{aligned}$$

where the $L_{S}$ and ${\mathcal {L}}_{S}$ is the clustering label and ground truth of sequence *S* respectively, and the accuracy, precision and recall are also calculated as the fraction of sequences correctly clustered against the ground truth [49], while $MI({\mathcal {C}},{\mathcal {G}})=\sum _{i=1}^{K}\sum _{j=1}^{K}\left(\frac{|C_{i}\cap G_{j}|}{N}\right)\log \left(\frac{N\times |C_{i}\cap G_{j}|}{|C_{i}|\times |G_{j}|}\right)$ is the mutual information, and $H({\mathcal {C}})=-\sum _{k=1}^{K}\left(\frac{|C_{k}|}{N}\right)\log \left(\frac{|C_{k}|}{N}\right)$ is the entropy. In fact, all those measures are derived from clustering results whose cluster number is the same as the actual class number, which are used to reveal how well the clustering results match with the actual class distribution. Larger value means better clustering performance.

### Results and analysis

To show the performance of DMSC, three performance metrics accuracy, F1-measure and NMI obtained on the test datasets by our model and the baselines are demonstrated in Tables 2, 3 and 4, respectively. It is easy to see that DMSC shows much better performance than that of the baselines on the six test datasets. DMSC achieves an improvement of 6.3%, 7.0% and 14.6% on average than that of second-place SMCD model on accuracy, F1-measure and NMI on all the test dataset, respectively. The significant improvement on the NMI means that DMSC outperforms the baselines in terms of the clustering quality and stability.

For the accuracy in Table 2, DMSC achieves the highest accuracy overall on the six test datatsets with average value is 91.7%; 10.6%, 6.6% and 4.8% higher than the second-place SMCD model on the protein, robot and pioneer dataset, and also betters the second-place SCS model by 3.2%, 3.3% and 3.0% on the pattern dataset, RNA and speech dataset respectively. In terms of the clustering quality and stability performance: the F1-measure in Table 3 and NMI in Table 4. It can be readily concluded that DMSC also outperforms the other baselines. DMSC earns the highest F1-measure and NMI value on all test datasets, especially for pioneer and robot dataset, where it achieves highest NMI over the second-place SMCD with a significant improvement of 24.5% and 13.5%, while yields 9.3% and 7.2% higher than that of the second-place SCS model on RNA and speech dataset. In short, DMSC achieves better performance over other baselines in terms of the clustering quality and stability based on the accuracy, F1-measure and NMI on the six test datasets.

**Table 2:** Comparision of performance in terms of accuracy

	Protein	RNA	Speech	Robot	Pioneer	Pattern	Average.
SigClust	0.723	0.689	0.831	0.648	0.638	0.713	0.707
SCS	0.914	0.861	0.834	0.835	0.793	0.838	0.846
CLUSEQ	0.827	0.603	0.835	0.739	0.731	0.754	0.745
WCPD	0.890	0.848	0.850	0.808	0.821	0.814	0.839
SMCD	0.924	0.829	0.878	0.859	0.825	0.809	0.854
DMSC	**0.962**	**0.893**	**0.918**	**0.925**	**0.931**	**0.871**	**0.917**

The best results are shown in bold

**Table 3:** Comparision of performance in terms of F1-measure

	Protein	RNA	Speech	Robot	Pioneer	Pattern	Average.
SigClust	0.386	0.706	0.832	0.647	0.637	0.611	0.637
SCS	0.920	0.861	0.861	0.834	0.792	0.745	0.836
CLUSEQ	0.598	0.532	0.843	0.738	0.730	0.652	0.685
WCPD	0.900	0.851	0.849	0.795	0.810	0.669	0.801
SMCD	0.928	0.830	0.877	0.858	0.824	0.704	0.837
DMSC	**0.964**	**0.894**	**0.917**	**0.924**	**0.930**	**0.810**	**0.907**

The best results are shown in bold

**Table 4:** Comparision of performance in terms of NMI

	Protein	RNA	Speech	Robot	Pioneer	Pattern	Average.
SigClust	0.157	0.332	0.658	0.243	0.207	0.368	0.346
SCS	0.702	0.651	0.801	0.355	0.421	0.609	0.589
CLUSEQ	0.451	0.435	0.702	0.422	0.421	0.471	0.494
WCPD	0.659	0.650	0.764	0.453	0.301	0.565	0.570
SMCD	0.721	0.619	0.789	0.481	0.501	0.552	0.601
DMSC	**0.838**	**0.744**	**0.873**	**0.616**	**0.755**	**0.658**	**0.747**

The best results are shown in bold

As we can see the results in Table 2, 3 and 4, SigClust achieves the lowest performance on all the test datasets due to the fact that it utilizes fixed-length patterns as signatures for sequence representation, assuming the pattern with fixed length is not realistic, since the chronological dependent structures or patterns varies differently in practice. SCS allows mismatching with fixed n-gram patterns, which allows to discover grams in variable length to make use of the sub-frequent patterns within n-gram for sequence representation. Thereby, it is reasonable that SCS outperforms SigClust. The CLUSEQ can detect variable length patterns by PST but suffers from underfitting that ignores the statistical information of individual sequences so that CLUSEQ shows better performance than that of SigClust but is inferior to SCS due to the variable length patterns and statistical information underlying sequences.

However, the WCPD model takes into account both the frequent consecutive patterns of variable length and the statistical information of individual sequences, but loss the information hidden in sparse patterns, which suffers from underfitting or overfitting because of the improper support threshold. Both of them thus show good performance on the test dataset which are better than CLUSEQ, but still inferior to DMSC due to the fact that they can not take advantage of the statistical information in sub-frequent patterns. SMCD, a Markov model, uses data compression technique to clustering the possible preceding subsequences with same length into invariance classes, which are used as the patterns of a fixed order Markov model, to predict the next categorical value for generative probability of a given sequence. It can take the information from the sub-frequent patterns into account, but still suffers from both the issue of fixed length pattern and underfitting (frequent consecutive patterns *GTGT* are represented by pattern $G*GT$ or $GT*T$ as argued above), which may explain the reason why SMCD performs better than others, but is also inferior to DMSC. As for DMSC, it neither suffers from overfitting nor underfitting, which not only retains the information hidden in consecutive patterns, but also makes use of the statistical information underlying sub-frequent patterns by the form of sparse pattern during the pattern detection process. All in all, the results also prove that DMSC outperforms the other baselines with respect to the performance metric of accuracy, F1-measure and NMI in Tables 2, 3 and 4 respectively.

### Scalability analysis

DMSC is scalable to the number of sequences and average length of the sequences, and the time complexity can be analysed by three parts of DMSC. The first part is data vectorization. The time complexity of this phase is mainly about of vectorizing all sequence in the dataset into an $\mathbf {MVS}$ matrix. The time of vectorizing sequence $S=s_{1}\ldots s_{n}$ to an $\mathbf {MVS}$ is ${\mathcal {O}}(n|\Sigma |+|\Sigma |^{2})$. Thus, the time complexity of sequence vectorization is ${\mathcal {O}}(N{\overline{n}}|\Sigma |+N|\Sigma |^{2})$, where ${\overline{n}}$ is the average length of the sequences in dataset, and $\Sigma$ is the number of categorical value set. The second part is initialization. The time complexity of this phase can be divided into two part: one is time complexity of PCA on the $\mathbf {MVS}$ matrix: ${\mathcal {O}}(N|\Sigma |R)$, where $R\le \sqrt{\min (n,|\Sigma |^{2})}$. The other is optimization by Chi-square distance ${\mathcal {O}}({\mathcal {I}}NM\log K)$, where ${\mathcal {I}}$ is the average number of iterations for the whole optimization process, *M* is the dimension of $\mathbf {MVS}$ after processed by PCA and *K* number of final clusters. Thus, the complexity of initialization is ${\mathcal {O}}(N|\Sigma |R+{\mathcal {I}}NM\log K)$. The final part is the clustering refinement. The time complexity of this phase also includes three part. The first part is the construction of SPD: ${\mathcal {O}}(N{\overline{n}}L|\Sigma |)$, where *L* is the maximal depth of SPD, the second is to detect patterns from SPD: ${\mathcal {O}}(|\Sigma |^{l})$, where $l \le L$ is the average length of pattern and involves the number of wildcards in the pattern. The final is clustering refinement, updating model parameters for one iteration is ${\mathcal {O}}(|C_{1}||\Sigma |^{l})$, the complexity of clustering refinement itself is ${\mathcal {O}}(N{\overline{n}}L|\Sigma |+{\mathcal {I}}NM\log K)$. Consequently, the time complexity of DMSC is linear with number of sequence *N* and sub-linear in terms of the average sequence length ${\overline{n}}$.

**Fig. 4:**
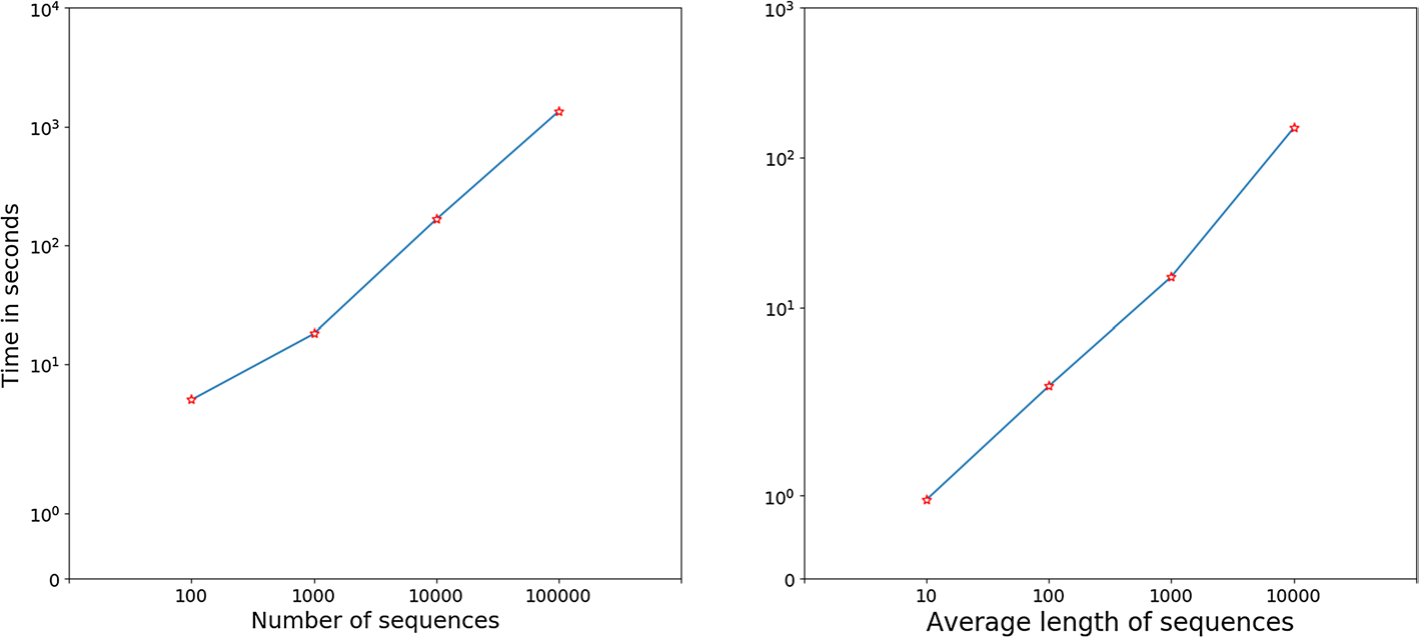
The time efficiency of DMSC with respect to the number of sequences and average sequence length

To examine the scalability of DMSC with respect to number of sequences and average length of sequences, we generated eight datasets with two class from two RNA families, and each class shares the same number of sequences. We use four datasets with size of $10^{2}$, $10^3$, $10^4$ and $10^5$ whose sequence length is 100 for the scalability of sequence numbers, while the rest four datasets with 1000 sequences whose average length sequences are 10, $10^2$, $10^3$ and $10^4$, respectively, which are used to test scalability of the average sequence length sequences. The running time are shown in Fig. 4. It can be clearly seen that the running time of DMSC increases linearly with respect to the number of sequences and the average length of sequences, respectively.

### Further analysis

Despite the performance against the baselines, we also present a in-depth analysis to investigate whether the sparse pattern is helpful for the clustering analysis or not by experiments. We have done a series of experiment to explore above question in the following two domains:

- SPD method can rich in pattern structure and cluster characterization?
- Sparse pattern can really improve the clustering performance?

#### Pattern richness and cluster characterization analysis

As for the pattern richness, we use SPD to detect the patterns from each cluster on the RNA dataset compared with consecutive detection methods. However, apart from the frequent consecutive patterns, SPD also detected the sparse patterns from each cluster, and top five frequent sparse patterns are shown in Table 5. What is most interesting is that these unique sparse patterns detected from one cluster can not be found in another cluster, which means that they are never co-occurred in two clusters and can be used to characterize the properties of cluster in view point of knowledge representation for clustering analysis. However, we will not focus on the biological analysis of these detected patterns like $$UGGU**U$$ in cluster $$C_{3}$$, and that will a part of our future work to investigate in the near future.

**Table 5:** Examples of sparse patterns on RNA dataset

Cluster	Top five sparse patterns of each cluster
$$C_{1}$$	$$CAA*U$$, $$GAA*C$$, $$GCA*C$$, $$GGUUC*A*U$$, $$GUU*G$$
$$C_{2}$$	$$CCA*C*A$$, $$GGGG*U$$, $$GACG*G$$, $$AAGC*U$$, $$AAAC*A$$
$$C_{3}$$	$$GGUC*G$$, $$UGGG*U$$, $$GUGG*G$$, $$UGGU**U$$, $$UGGC*G$$

**Fig. 5:**
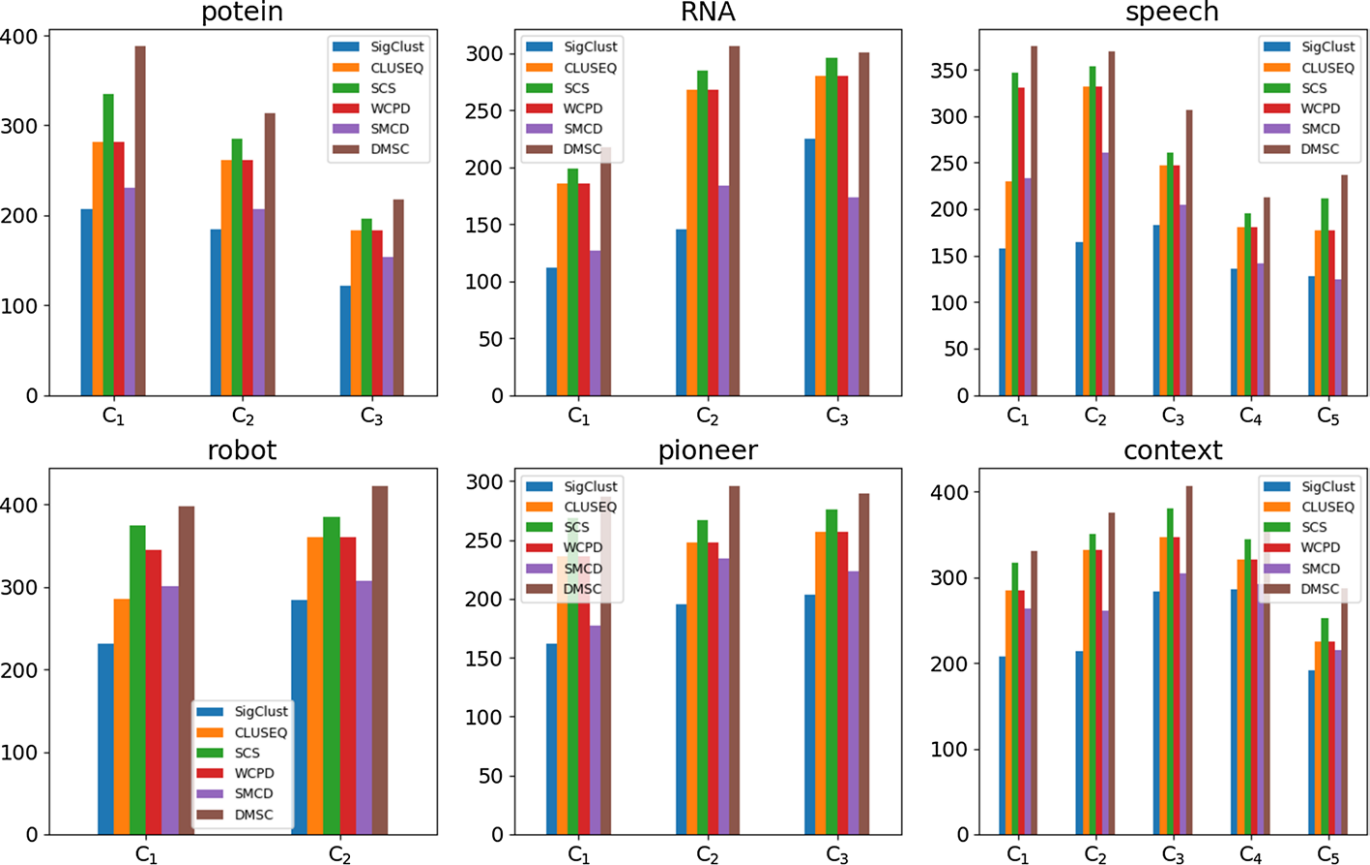
The number of patterns detected by six baselines from each clusters on the six test datasets, and our model has more patterns than other baselines, which demonstrates the pattern richness of DMSC for sequence clustering analysis and knowledge representation

We also compared the detected patterns using for clustering against the baselines from each cluster on test datasets. The patterns detected from the final clustering results are shown in Fig. 5. We thus can learn that DMSC has the largest number of patterns due to the fact that SPD detects sparse patterns from sub-frequent patterns by containing wildcards, while also remains the frequent consecutive patterns simultaneously. It can be obviously concluded that such pattern richness is of great help for sequence analysis and knowledge representation in view point of a statistic model. Moreover, these sparse patterns are capable of taking advantage of the statistical information underlying the sub-frequent patterns, a common sub-optimization problem of pattern detection of sequence mining, which may explain the reason why DMSC has the largest number of patterns and outperforms the other baselines in terms of clustering results.

#### Clustering improvement of sparse pattern analysis

**Fig. 6:**
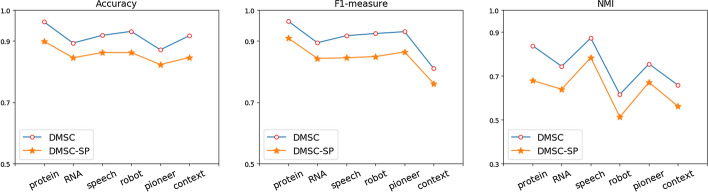
Three performance metrics of DMSC and DMSC-SP on test datasets. DMSC-SP, the DMSC without sparse pattern, is designed to use only consecutive pattern to validate of the effects of sparse patterns for clustering analysis

As for the clustering performance improvement, we experiment the DMSC without the sparse patterns on test datasets, named DMSC-SP. Such implementation can directly validate the usefulness of the sparse patterns whether they can improve the performance of clustering result or not. As the results shown in Fig. 6, it can easily be concluded that the clustering performance of DMSC on the test dataset outperforms that of DMSC-SP without sparse patterns, as results of DMSC is obviously higher than that of DMSC-SP. Actually, DMSC improves significantly on three performance metrics on text dataset, around 6.0% improvement on accuracy, F1-measure, and NMI on average, which validates that the sparse pattern is of great importance to improve the performance of clustering. Thereby, we can make sure that those sparse patterns detected by SPD can be of great help to characterizing clusters for clustering analysis.

In summary, DMSC, a based statistical model for categorical sequence clustering, not only takes the consecutive patterns with variable length and statistical information, such as the number of sequence in each cluster and sequence length, into account, but also makes use of the information underlying in sparse patterns detected from the sub-frequent patterns for the clustering analysis. SPD is a powerful pattern detector in terms of the pattern richness in the domain of pattern recognition, since it not only detects consecutive pattern, but also retains the possibility of discovering frequent sparse patterns from the sub-frequent patterns. Moreover, it also avoids overfitting and underfitting by retaining the some sub-frequent patterns, thus sidestepping the sub-optimal pattern detection problem, a common limitation of pattern mining. Finally, the experimental results on real-world datasets also exhibit the superiority of the DMSC over a number of state-of-the-art competitors.

## Conclusion

In this paper, we propose a Dynamic order Markov model (DOMM) that is capable of dealing with the sparse patterns and handling the length of the sparse patterns adaptively, i.e. allowing variable length pattern, which allows deriving a similarity measure between a sequence and a set of sequences/cluster. To implement DOMM, we propose a sparse pattern detector (SPD) to discover sparse and consecutive patterns based on the PST containing wildcards, which can take advantage of significant statistical information, specially the information veiled by the noise in the form of sparse pattern. We then develop a divisive clustering algorithm, named DMSC, for Dynamic order Markov model for Categorical Sequence Clustering. Experimental results on real-world datasets demonstrate the promising performance of the proposed model.

Our future work may focus on the following aspects: on one hand, we would like to improve the performance of the for sparse pattern detection, and investigate how this kind of sparse patterns would be useful for knowledge discovery and model representation in the field of bioinformatics, such as the mutation patterns and the evolution of biological sequence. On the other hand, we would like to apply the SPD on financial data or event data to detect complex patterns for decision making or behavior analysis. In short, we see the significant challenges for our future work, but we are confident that the proposed method has great potential in real applications.

## Data Availability

Protein dataset is available at: http://www.uniprot.org/. RNA dataset is available at: http://rfam.xfam.org. Speech dataset is available from the corresponding author on reasonable request. Robot dataset is available at: https://archive.ics.uci.edu/ml/datasets/Wall-Following+Robot+Navigation+Data. Pioneer dataset is availabe at: https://archive.ics.uci.edu/ml/datasets/Pioneer-1+Mobile+Robot+Data. Pattern dataset is available at http://www.cis.hut.fi/jhimberg/patterndata/index.shtml.

## Abbreviations

DOMM: Dynamic order Markov model
SPD: Sparse pattern detector
PST: Probability suffix tree
DMSC: Dynamic order Markov model for categorical sequence clustering
DMSC-SP: DMSC without sparse pattern
MVS: Markov vector of sequence
PCA: Principal component analysis
NMI: Normalized mutual information

## Footnotes

1 A pattern is statistically significant whose occurrence is no less than the user-defined support threshold $\tau$, otherwise the pattern is or sub-frequent or non-statistically significant.

2 In this paper, the terminology of context and pattern are used interchangeably.

3 The * denotes a wildcard. To compared with the consecutive pattern, we call the pattern containing wildcard/s as sparse pattern. Sparse pattern $GT*T$ consists of sub-frequent pattern *GTAT* and *GTIT*, and the wildcard * only matches with I, A.

## References

[1] Agrawal R, Srikant R. Mining sequential patterns. In: Data engineering, 1995. Proceedings of the eleventh international conference on, 1995. IEEE. p. 3–14.

[2] Mobasher B, Dai H, Luo T, Nakagawa M. Using sequential and non-sequential patterns in predictive web usage mining tasks. In: Data Mining, 2002. ICDM 2003. Proceedings. 2002 IEEE international conference on, 2002. IEEE. p. 669–72.

[3] Petridou SG, Koutsonikola VA, Vakali AI, Papadimitriou GI. A divergence-oriented approach for web users clustering. In: International conference on computational science and its applications. Springer; 2006. p. 1229–38.

[4] Gebser M, Guyet T, Quiniou R, Romero J, Schaub T. Knowledge-based sequence mining with asp. In: IJCAI 2016-25th international joint conference on artificial intelligence, AAAI. 2016. p. 8.

[5] He Z, Zhang S, Wu J (2019). Significance-based discriminative sequential pattern mining. Expert Syst Appl.

[6] Ibrahim R, Shafiq MO (2019). Detecting taxi movements using random swap clustering and sequential pattern mining. J Big Data.

[7] Lin Z, Lanchantin J, Qi Y. Must-cnn: a multilayer shift-and-stitch deep convolutional architecture for sequence-based protein structure prediction. 2016. arXiv preprint arXiv:1605.03004.

[8] Katoh K, Rozewicki J, Yamada KD (2019). Mafft online service: multiple sequence alignment, interactive sequence choice and visualization. Brief Bioinform.

[9] Fournier-Viger P, Lin JC-W, Kiran RU, Koh YS, Thomas R (2017). A survey of sequential pattern mining. Data Sci Pattern Recogn.

[10] Rathee S, Kashyap A (2018). Streamaligner: a streaming based sequence aligner on apache spark. J Big Data.

[11] Yuan L, Wang W, Chen L (2019). Two-stage pruning method for gram-based categorical sequence clustering. Int J Mach Learn Cybern.

[12] Wicker N, Perrin GR, Thierry JC, Poch O (2001). Secator: a program for inferring protein subfamilies from phylogenetic trees. Mol Biol Evol.

[13] Watson JD, Laskowski RA, Thornton JM (2005). Predicting protein function from sequence and structural data. Curr Opin Struct Biol.

[14] Borges J, Levene M (2007). Evaluating variable-length markov chain models for analysis of user web navigation sessions. IEEE Trans Knowl Data Eng.

[15] Ki Y, Yoon JW. Pd-fds: purchase density based online credit card fraud detection system. In: KDD 2017 workshop on anomaly detection in finance. 2018. p. 76–84.

[16] Altschul SF, Gish W, Miller W, Myers EW, Lipman DJ (1990). Basic local alignment search tool. J Mol Biol.

[17] Needleman SB, Wunsch CD (1970). A general method applicable to the search for similarities in the amino acid sequence of two proteins. J Mol Biol.

[18] Liu L, Ho Y-K, Yau S (2006). Clustering DNA sequences by feature vectors. Mol Phylogenet Evol.

[19] Xiong T, Wang S, Jiang Q, Huang JZ (2014). A novel variable-order markov model for clustering categorical sequences. IEEE Trans Knowl Data Eng.

[20] Chappell T, Geva S, Hogan J. K-means clustering of biological sequences. In: Proceedings of the 22nd Australasian document computing symposium, ACM. 2017. p. 2.

[21] Ching WK, Fung ES, Ng MK (2004). Higher-order markov chain models for categorical data sequences. Naval Res Logist.

[22] Xu J, Wickramarathne TL, Chawla NV (2016). Representing higher-order dependencies in networks. Sci Adv.

[23] Garofalakis MN, Rastogi R, Shim K. Spirit: sequential pattern mining with regular expression constraints. In: VLDB 1999.

[24] Zaki MJ. Sequence mining in categorical domains: incorporating constraints. In: Proceedings of the ninth international conference on information and knowledge management, ACM. 2000. p. 422–9.

[25] Pei J, Han J, Mortazavi-Asl B, Pinto H, Chen Q, Dayal U, Hsu M-C. Prefixspan: mining sequential patterns efficiently by prefix-projected pattern growth. In: Icccn, IEEE. 2001. p. 0215 .

[26] Ji X, Bailey J, Dong G (2007). Mining minimal distinguishing subsequence patterns with gap constraints. Knowl Inf Syst.

[27] Yang J, Wang W. Cluseq: efficient and effective sequence clustering. In: Data engineering, 2003. Proceedings. 19th international conference on, IEEE. 2003; p. 101–12.

[28] Kelil A, Wang S, Jiang Q, Brzezinski R (2010). A general measure of similarity for categorical sequences. Knowl Inf Syst.

[29] Fowkes J, Sutton C. A subsequence interleaving model for sequential pattern mining. In: Proceedings of the 22nd ACM SIGKDD international conference on knowledge discovery and data mining, ACM. 2016. p. 835–44.

[30] Steinegger M, Söding J (2018). Clustering huge protein sequence sets in linear time. Nat Commun.

[31] Mitra S, Sastry P. Summarizing event sequences with serial episodes: a statistical model and an application. 2019. arXiv preprint arXiv:1904.00516.

[32] Martin DE (2019). Minimal auxiliary markov chains through sequential elimination of states. Commun Stat-Simul Comput.

[33] Amer AA, Abdalla HI (2020). A set theory based similarity measure for text clustering and classification. J Big Data.

[34] Park S, Kang I, Shin C (2021). MicroRNA clustering on the biogenesis of suboptimal microRNAs. Appl Biol Chem.

[35] Altschul SF, Madden TL, Schäffer AA, Zhang J, Zhang Z, Miller W, Lipman DJ (1997). Gapped blast and psi-blast: a new generation of protein database search programs. Nucleic Acids Res.

[36] Xie F, Wu X, Hu X, Gao J, Guo D, Fei Y, Hua E. Sequential pattern mining with wildcards. In: Tools with artificial intelligence (ICTAI), 2010 22nd IEEE international conference on, IEEE; vol. 1. 2010. p. 241–7.

[37] Xiong J, Jääskinen V, Corander J (2016). Recursive learning for sparse markov models. Bayesian Anal.

[38] Martin DE (2020). Distributions of pattern statistics in sparse Markov models. Ann Inst Stat Math.

[39] Hu J, Turin W, Brown MK (1997). Language modeling using stochastic automata with variable length contexts. Comput Speech Lang.

[40] Jääskinen V, Xiong J, Corander J, Koski T (2014). Sparse markov chains for sequence data. Scand J Stat.

[41] Rissanen J (1983). A universal data compression system. IEEE Trans Inf Theory.

[42] Xiong T, Wang S, Jiang Q, Huang JZ. A new markov model for clustering categorical sequences. In: 2011 IEEE 11th international conference on data mining, IEEE. 2011; p. 854–63.

[43] Ramoni M, Sebastiani P, Berthold M, Hand DJ (1999). Bayesian methods. Intelligent data analysis.

[44] Loiselle S, Rouat J, Pressnitzer D, Thorpe S. Exploration of rank order coding with spiking neural networks for speech recognition. In: Neural networks. Proceedings. 2005 IEEE international joint conference on, IEEE; vol. 4. 2005. p. 2076–80.

[45] Zhou C, Cule B, Goethals B (2015). Pattern based sequence classification. IEEE Trans Knowl Data Eng.

[46] Fradkin D, Mörchen F (2015). Mining sequential patterns for classification. Knowl Inf Syst.

[47] Flanagan JA, Mäntyjärvi J, Himberg J. Unsupervised clustering of symbol strings and context recognition. In: 2002 IEEE international conference on data mining, 2002. Proceedings. 2002. p. 171–8.

[48] García S, Fernández A, Luengo J, Herrera F (2010). Advanced nonparametric tests for multiple comparisons in the design of experiments in computational intelligence and data mining: Experimental analysis of power. Inf Sci.

[49] Banerjee A, Krumpelman C, Ghosh J, Basu S, Mooney RJ. Model-based overlapping clustering. In: Proceedings of the eleventh ACM SIGKDD international conference on knowledge discovery in data mining, ACM. 2005. p. 532–7.

